# Intercellular crosstalk between cancer cells and cancer-associated fibroblasts via exosomes in gastrointestinal tumors

**DOI:** 10.3389/fonc.2024.1374742

**Published:** 2024-02-23

**Authors:** Longyang Cao, Hong Ouyang

**Affiliations:** Department of Gastroenterology, The First Peoples’ Hospital of Hangzhou Linan District, Hangzhou, China

**Keywords:** gastrointestinal (GI) tumors, cancer-associated fibroblasts (CAFs), exosomes, tumor microenvironment (TME), review

## Abstract

Gastrointestinal (GI) tumors are a significant global health threat, with high rates of morbidity and mortality. Exosomes contain various biologically active molecules like nucleic acids, proteins, and lipids and can serve as messengers for intercellular communication. They play critical roles in the exchange of information between tumor cells and the tumor microenvironment (TME). The TME consists of mesenchymal cells and components of the extracellular matrix (ECM), with fibroblasts being the most abundant cell type in the tumor mesenchyme. Cancer-associated fibroblasts (CAFs) are derived from normal fibroblasts and mesenchymal stem cells that are activated in the TME. CAFs can secrete exosomes to modulate cell proliferation, invasion, migration, drug resistance, and other biological processes in tumors. Additionally, tumor cells can manipulate the function and behavior of fibroblasts through direct cell-cell interactions. This review provides a summary of the intercellular crosstalk between GI tumor cells and CAFs through exosomes, along with potential underlying mechanisms.

## 1 Introduction

Gastrointestinal (GI) tumors, such as gastric cancer (GC), colorectal cancer (CRC), esophageal cancer (EC), hepatocellular carcinoma (HCC), and pancreatic cancer (PC), have become a significant health threat in recent years. EC is a cancerous tumor that originates from the esophagus and presents symptoms such as choking sensation, foreign body sensation, retrosternal pain, or difficulty swallowing. It can be categorized into different subtypes, with esophageal squamous cell carcinoma (ESCC) being the most common type in China and esophageal adenocarcinoma (EAC) more prevalent in Western countries (1). ESCC and EAC are different in terms of pathogenesis, molecular biology, and prognosis. The causes of ESCC are related to factors like smoking, alcohol consumption, nitrite food intake, poor dietary habits, and genetic susceptibility (2, 3). While EAC is associated with obesity, gastroesophageal reflux disease, and Barrett’s esophagus (4, 5). EC is characterized by local invasion, rapid progression, high recurrence rate, and poor survival prognosis (6). Although therapies such as surgical resection, radiotherapy and immunotherapy have made great advance in recent years, the overall survival (OS) rate of EC patients remains poor (7). Gastric cancer (GC) is a malignant tumor that originates in the stomach lining and is characterized by high morbidity and mortality rates (8, 9). Factors like helicobacter pylori (Hp) infection, poor dietary habits, smoking, alcohol consumption, psychological factors, and genetics are closely linked to the advancement of GC (10). Currently, minimally invasive surgery combined with radiotherapy, immunotherapy and targeted therapy has made significant progress in GC (11, 12), but the 5-year OS remains unsatisfactory. Colorectal cancer (CRC) is a deadly tumor that originates in the large intestine and the incidence, morbidity, and mortality rates of it have been increasing over the years (13). The progress of CRC is related to various factors such as a high-fat diet, low dietary fiber intake, obesity, and smoking (14, 15), and the clinical manifestations are usually characterized by abdominal pain, abdominal distension, intestinal obstruction, blood in the stools, and emaciation without any obvious triggers (16). Treatment modalities for CRC include surgery, chemotherapy, radiotherapy, and targeted therapy (17, 18). Hepatocellular carcinoma (HCC) can be divided into primary HCC and secondary HCC. Primary HCC originates from liver cells, while secondary HCC refers to metastasis from other organs to the liver (19, 20). Long-term alcohol consumption, diabetes or obesity-related non-alcoholic fatty liver, HBV or HCV infection, and cirrhosis of any etiology are the triggers of HCC (21). Treatment options for early-stage HCC include surgical resection, liver transplantation, and local ablation, while transarterial chemoembolization (TACE) or systemic therapy refer to the patients with advanced-stage HCC (22, 23). Pancreatic cancer (PC) originates from the pancreatic ductal epithelium and follicular cells. It is highly lethal and often diagnosed at an advanced stage due to the lack of early detection methods and clinical indicators (24). Less than 20% of patients have locally curable tumors at the time of diagnosis (25). Even after curative surgery, localized and metastatic recurrence is common in the early stages of disease progression (26, 27).

Distant metastasis is a complex process in cancer where cancer cells spread to other organs. Fibroblasts and cytokines in the target organ create a favorable environment for cancer cell colonization. Cancer cells undergo epithelial mesenchymal transition (EMT) and break through the basement membrane to enter the circulation (28, 29). These circulating tumor cells then colonize specific environments to form metastatic tumors. Cancer-associated fibroblasts (CAFs) are a significant component of the tumor microenvironment (TME) and play a crucial role in various stages of metastasis (30). Beyond that, CAFs can secrete large amounts of metalloproteinases and fibrin activators to directly degrade the extracellular matrix (ECM) (31, 32). Besides, CAFs can secrete cytokines that promote EMT in cancer cells and produce enzymes that degrade the ECM. They also exert physical force on tumor cells through cell adhesion molecules, facilitating collective invasion (33, 34). Additionally, CAFs participate in pre-metastatic niches and promote the colonization of metastatic cancer cells (35, 36).

Exosomes are a newly discovered sort of extracellular vesicles (EVs) in recent years, which are flat, spherical, or cupped, and are viewed as a third mode of intercellular information transfer (37). Exosomes originate from the internal outgrowth of the cell membrane and are liberated outside the cell after selectively wrapping some cytoplasmic components such as lipids, proteins, and nucleic acids. Hence, exosomes relate to numerous pathological and physiological progresses in humans (38–40). Many researches have proved that exosomes are engaged in diversiform platform of malignant progression in GI tumors. For examples, exosomal circSHKBP1 promotes lethal progression of GC by inhibiting the miR-582-3p/HUR/VEGF signaling axis as well as regulating HSP90 degradation (41). Exosomal circLPAR1 plays an important biological role in CRC diagnosis and tumorigenesis by METTL3-eIF3h interaction and inhibition of BRD4 (42). Exosome-derived circCCAR1 significantly promotes CD8^+^ T cell dysfunction and anti-PD1 resistance in HCC (43). All above studies have proved that exosomes can promote lethal progression of GI tumors by delivering multiple pro-oncogenic factors in TME. Furthermore, exosomal interactions between CAFs and cancer cells have been implicated in tumorigenesis and progression. Exosomes derived from CAFs can affect the activation of certain signaling pathways in cancer cells, modulate gene expression, and contribute to chemoresistance. CAFs-derived LINC01614 can promote the activation of NF-κB by interacting with ANXA2 and p65, resulting in the up-regulation of glutamine transporter proteins SLC38A2 and SLC7A5, and contributing to the enhancement of glutamine influx in lung adenocarcinoma cells (44). Exosomal miR-146a-5p can upregulate SVEP1 in CAFs by enhancing the recruitment of the transcription factor YY1 and targets ARID1A and PRKAA2, thereby contributing to the stemness, chemoresistance to gemcitabine and cisplatin of bladder cancers (45). Exosomal miRNA-20a derived from CAFs can inhibit the PTEN/PI3K-AKT pathway thus stimulating the development and cisplatin resistance in non-small cell lung cancer (46). In this review, we summarized the role of exosomes in the interaction between GI tumors cells and CAFs and potential biological mechanism.

## 2 Overview of CAFs

CAFs are significantly different from normal fibroblasts (NFs) in terms of morphological features, biological and secretory function. CAFs are characterized by their large spindle-shaped morphology, irregular nuclei with noticeable notches, abundant cytoplasm, myofilaments, and an abundance of rough endoplasmic reticulum within the cytoplasm. CAFs have almost all the characteristics of normal fibroblasts, but they are more active than normal fibroblasts, proliferate faster, secrete more cytokines, matrix proteins and immunomodulatory factors to engage in the regulation of TME. Hence, they serve as an important role in tumorigenesis and advancement (32, 47). As a crucial component of the TME, CAFs contribute to inhibiting the malignant behavior of tumor cells through the secretion of various cytokines, growth factors, and ECM proteins (48, 49).

### 2.1 The origin of CAFs

**Figure 1:**
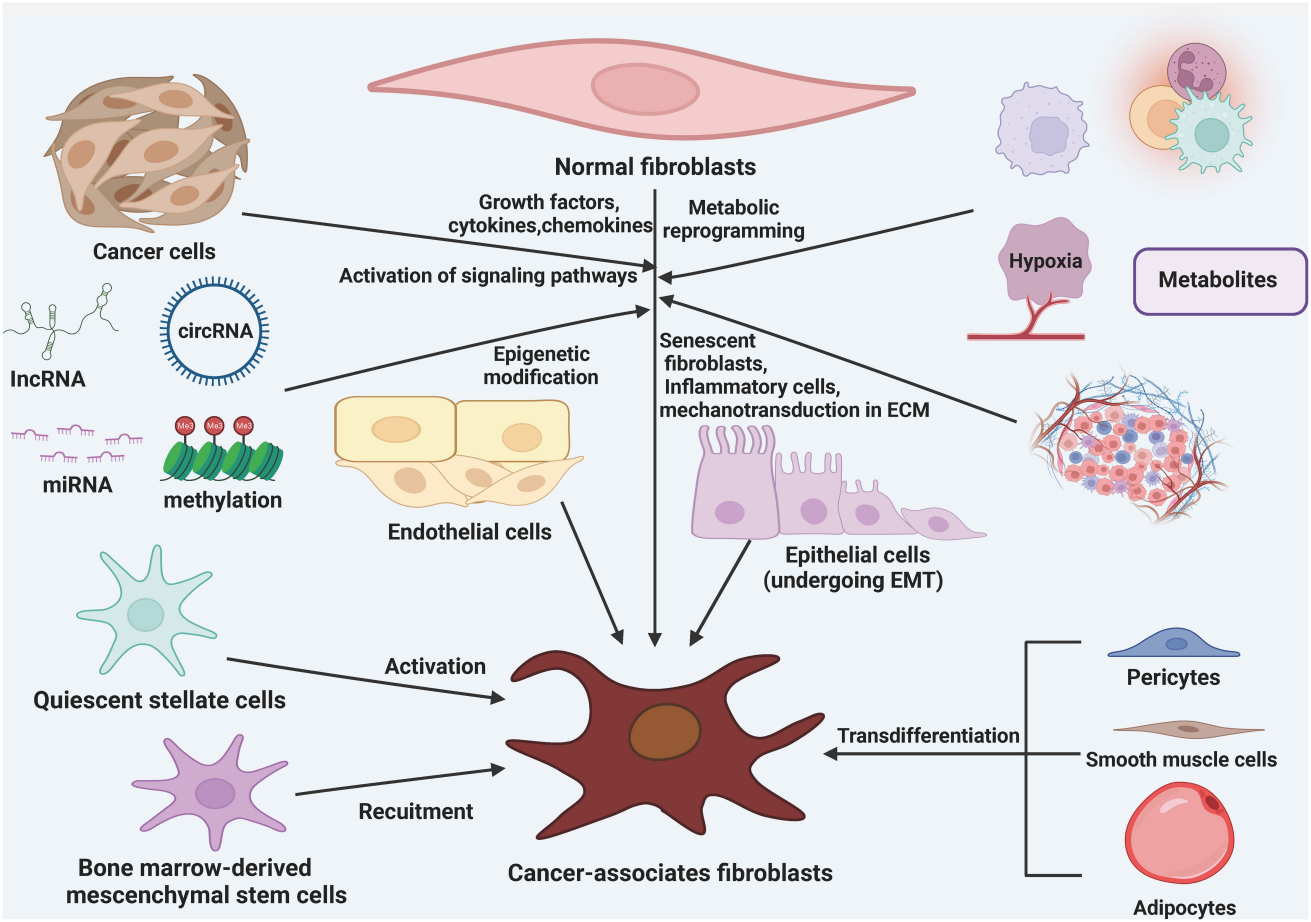
The origin of cancer-associated fibroblasts (CAFs). CAFs can be formed from a variety of cellular precursors through specific mechanisms. Multiple signaling pathways in tissue-resident NFs can be activated in response to stimulation by growth factors, cytokines, and chemokines, as well as epigenetic modifications, and can lead to the conversion of these cells into CAFs. in addition, metabolic reprogramming induced by metabolites derived from cancer cells, senescent fibroblasts, inflammatory cells, and mechanotransduction in the ECM also mediate the transformation of NFs into CAFs. BMSCs and quiescent stellate cells are also recruited and activated to become CAFs in response to growth factors, cytokines, and chemokines. In addition, adipocytes, pericytes, endothelial cells, epithelial cells, and smooth muscle cells are also converted to CAFs.

There are various theories regarding the origin of CAFs, but their exact origins are still not fully understood. It is currently believed that CAFs mainly arise from resident fibroblasts, bone marrow mesenchymal stem cells (BMSCs), adipocytes, pericytes, endothelial cells, epithelial cells, and smooth muscle cells (28, 50, 51) (**Figure 1**). Through the influence of growth factors, cytokines, chemokines, and epigenetic modifications, different signaling pathways in tissue resident normal fibroblasts can be activated and transformed into CAFs (52, 53). Metabolic reprogramming, induced by metabolic products from cancer cells, aging fibroblasts, inflammatory cells, and mechanical forces within the ECM, is another factor that contributes to the conversion of normal fibroblasts into CAFs. In various types of cancer tissues, resident fibroblasts present in the surrounding areas are an essential source of CAFs within the TME. Growth factors, cytokines, and chemokines can also recruit and activate BMSCs and stationary stellate cells, transforming them into CAFs (54–56). TGF-β1 has been found to induce endothelial cells to undergo a phenotypic transformation into fibroblasts. Additionally, adipocytes can promote fibroblast differentiation by directly interacting with tumor cells through adipose stem cells. Furthermore, pericytes, epithelial cells, and smooth muscle cells can also undergo conversion into CAFs (57, 58). 

### 2.2 The heterogeneity of CAFs

**Figure 2:**
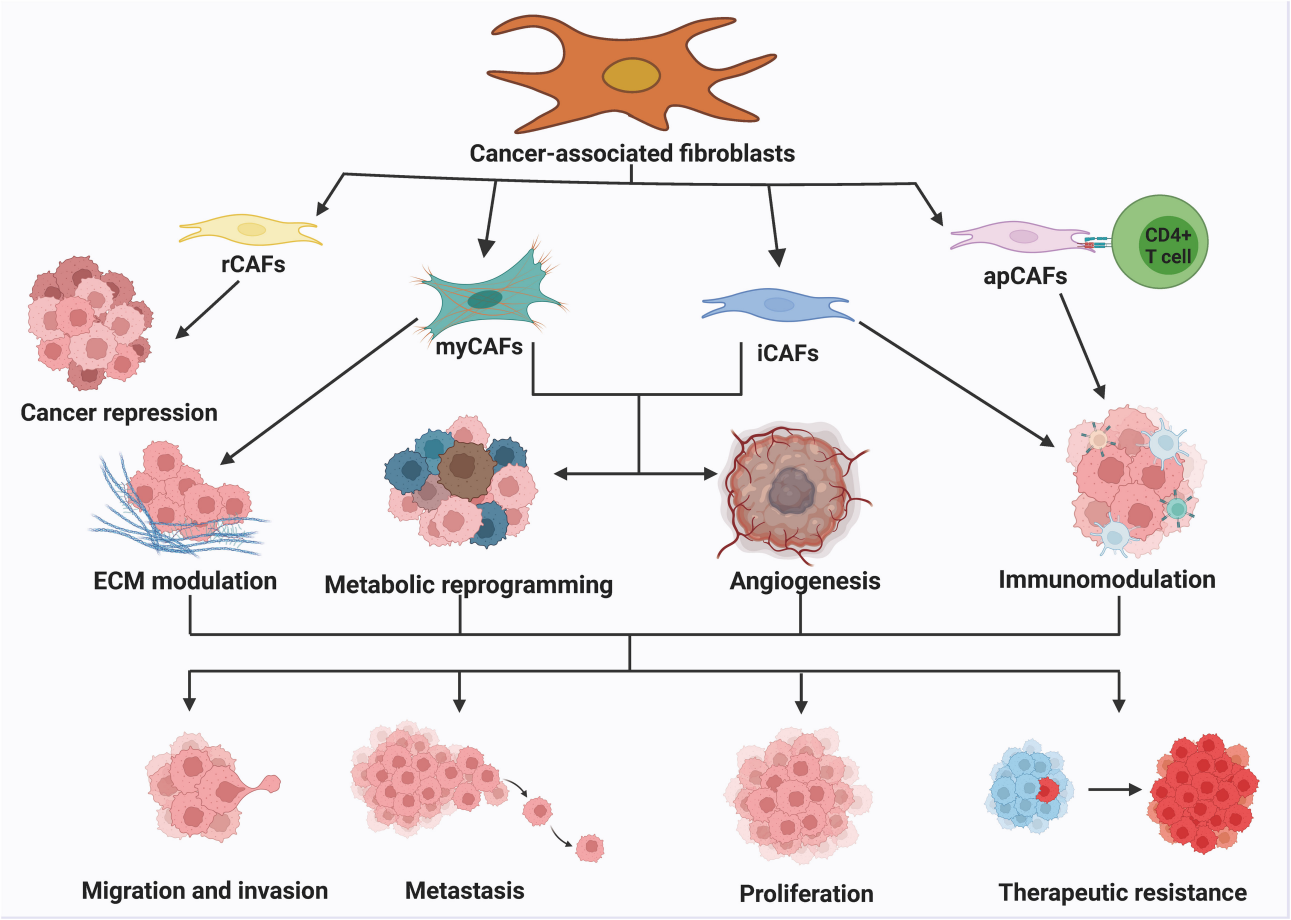
The classification and function of CAFs. The numerous routes of origin of CAFs lead to the high heterogeneity of CAFs. Generally, CAFs can be classified into rCAFs, myCAFs, iCAFs and apCAFs. rCAFs mainly play an oncogenic role. myCAFs promote ECM remodeling by synthesizing collagen and regulating mechanotransduction. iCAFs regulate the immune process by altering secretory properties. apCAFs activate CD4+ T cells in an antigen-specific manner. myCAFs, iCAFs and apCAFs together can significantly activate CD4+ T cells. The combination of myCAFs, iCAFs and apCAFs significantly promotes cancer cell proliferation, migration, invasion, metastasis and drug resistance, thereby contributing to the progression of cancer.

The heterogeneity of CAFs can be attributed to the various pathways through which they originate. CAFs express different markers such as α-smooth muscle actin (α-SMA), fibroblast activating protein (FAP), tendon protein C, periosteal proteins, NG2 chondroitin sulfate proteoglycans, and platelet-derived growth factor α (PDGF-α) (59–62). FAP is commonly used in tumor therapy and diagnostic imaging and is involved in regulating the extracellular matrix, exhibiting pro-tumorigenic activities. α-SMA is used to evaluate the therapeutic function of targeted CAFs and promotes tumor angiogenesis by regulating cell proliferation and the transport of pro-angiogenic factors. Inhibition of α-SMA transport attenuates angiogenic capacity and thus inhibiting tumor progression. Other markers such as waveform protein, proline 4 hydroxylase, fibronectin, type I collagen, fibroblast-specific protein-1, and fibroblast surface proteins are used to characterize mesenchymal stromal cells, including CAFs. The existence of heterogeneity in CAFs is primarily due to differences in cellular phenotypes, which exhibit two characteristics: temporal (associated with tumor development stage) and spatial (distinct phenotypes in different areas of cancer tissues) (63, 64). CAFs can be categorized into reactive CAFs (rCAFs), myofibroblast CAFs (myCAFs), inflammatory CAFs (iCAFs), and antigen-presenting CAFs (apCAFs). The combined effect of myCAFs, iCAFs, and apCAFs obviously promote cancer cell proliferation, migration, invasion, metastasis, and drug resistance, ultimately contributing to cancer development (65, 66) (**Figure 2**). The role of CAFs in various aspects of cancer is well established. CAFs can promote cancer progression through different mechanisms. myCAFs can promote ECM remodeling by synthesizing collagen and regulating mechanical conduction. ICAFs can regulate the immune process of the body by altering their secretion characteristics. ApCAFs can activate CD4+T cells in an antigen-specific manner. The combined effect of myCAFs, iCAFs, and apCAFs can ultimately significantly promote the proliferation, migration, invasion, metastasis, and drug resistance of cancer cells, thereby promoting the development of cancer.

### 2.3 The biological function of CAFs in cancers

**Figure 3:**
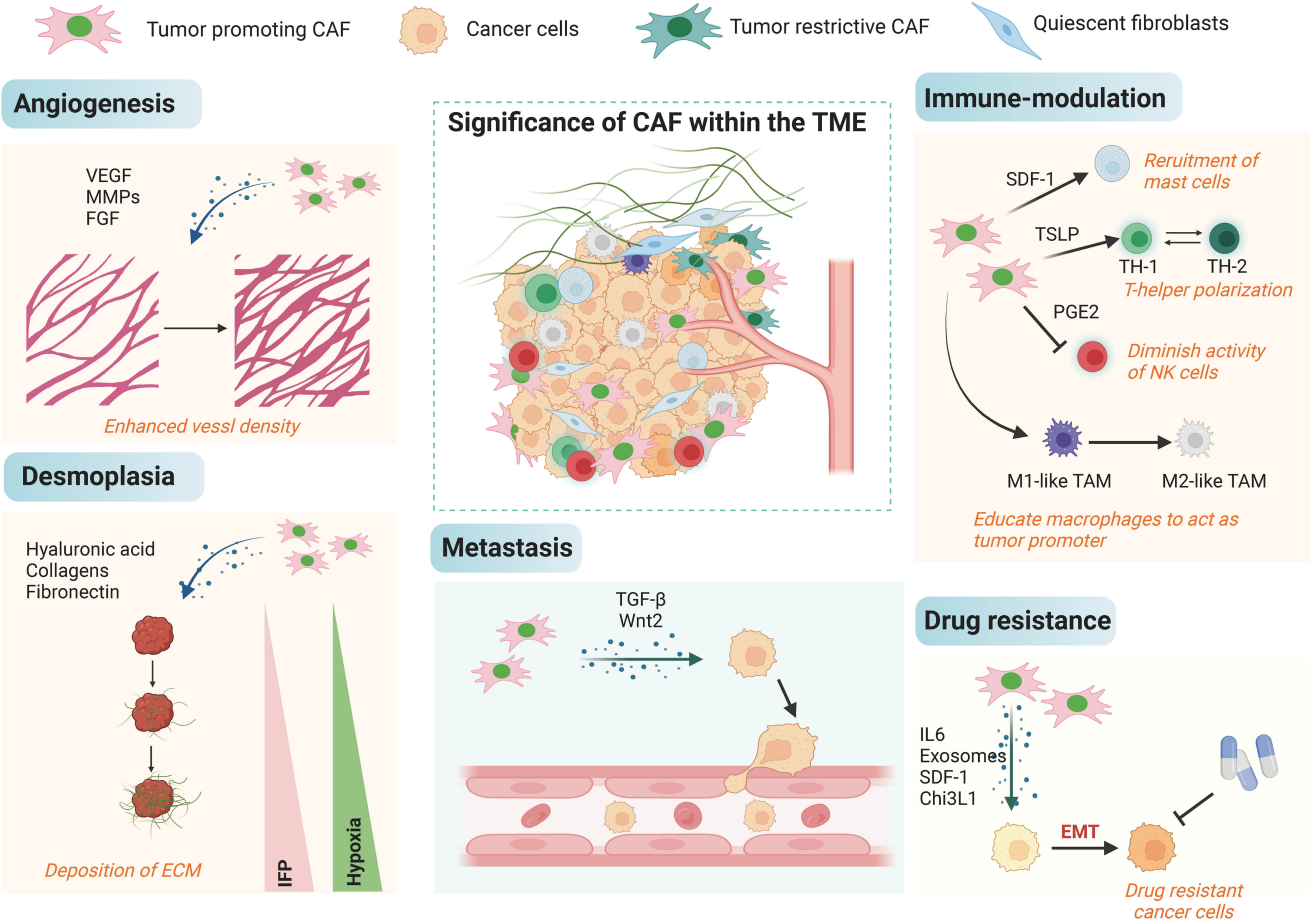
The biological roles of CAFs in cancer. The tumor microenvironment is complex, and CAFs can influence multiple processes in the tumor microenvironment such as angiogenesis, decalcification, hypoxia, immune regulation, and EMT in cancer cells by orchestrating the interactions of multiple cell types and processes, leading to metastasis and high drug resistance.

The function of CAFs in different aspects of cancers has been well established, and they could accelerate cancer progression through a variety of mechanisms (**Figure 3**). Most of the current studies support that CAFs primarily take a tumor-promoting function, and it is believed that targeting CAFs may be a future-proof tactics for cancer therapy. First, CAFs can promote tumor growth. CAFs are widely distributed in the tumor stroma and are in close contact with tumor cells, and it can directly mutual effect with tumor cells to accelerate tumor growth (67, 68). Besides, CAFs are the main source of growth factors, cytokines and exosomes in the TME, which can indirectly promote tumor growth (69–71). ITGB2-mediated metabolic switching of CAFs promotes oral squamous cell carcinoma (OSCC) proliferation through the oxidation of NADH in the mitochondrial oxidative phosphorylation system (72). Cancer metastasis is a multilevel procedure. Firstly, cancer cells break through the substrate membrane to migrate and invade into the ambient tissues. After endocytosis, dissemination and exocytosis, several surviving cancer cells will ectopically colonize other tissues, and ultimately form metastatic foci that are visible to the naked eye. CAFs have a crucial promotional function in the process of tumor metastasis (73, 74). Exosomes derived from CAFs miR-18b could accelerate breast cancer invasion and metastasis by regulating TCEAL7 (75). Resistance to cancer therapies usually result in tumor progression, and many studies have identified a function for stromal CAFs in tumor drug resistance. PDPN-positive CAFs can accelerate resistance to trastuzumab in HER2-positive breast cancer by holding back antibody-dependent NK cell-mediated cytotoxicity (76). Factors secreted by CAFs also affect other components of the TME, and they could do it according to diversity of immune cells, such as CD8^+^ T cells, regulatory T cells (Tregs), and macrophages, with immunomodulatory effects (77, 78). Most of the current studies suggest that the main role of CAFs is immunosuppression such as the immunosuppressive molecules IL-10, TGF-β and certain CXCL secreted by CAFs can significantly inhibit the proliferation and activation of T cells.

## 3 Overview of exosomes

EVs are tiny structures enclosed by lipid bilayers that are naturally released from different types of cells into the surrounding environment (79, 80). According to the size of extracellular vesicles, they can be further divided into exosomes (with a diameter of 50-100 nm, formed by multi vesicle exocytosis), microvesicles (with a diameter of 100-1000 nm, formed by plasma membrane sprouting), and apoptotic bodies (with a diameter of 1-5μm and released during cell death) (81, 82). Exosomes are secreted by a variety of cells and produced by coalition of intracellular multivesicular bodies with the cell membrane to be released out of the cell (83, 84). Exosomes could be acquitted by disparate form of cells for example macrophages, tumor cells, mesenchymal stem cells, epithelial cells, mast cells, endothelial progenitor cells and fibroblasts (85, 86). Exosomes are commonly distributed in a diversity of body fluids such as human urine, saliva, blood, cerebrospinal fluid, and bile (87, 88)and consist of biomolecules including proteins, lipids, and nucleic acids. The lipid bilayer of exosomes contains cholesterol, sphingolipids, and ceramides, which protect the biologically active components from degradation (89). The protein composition of exosomes includes membrane fusion proteins, proteins involved in the formation of EVs, heat shock proteins, myosin heavy chain class II proteins, integrins, and transmembrane proteins (90).

### 3.1 The biogenesis of exosomes

**Figure 4:**
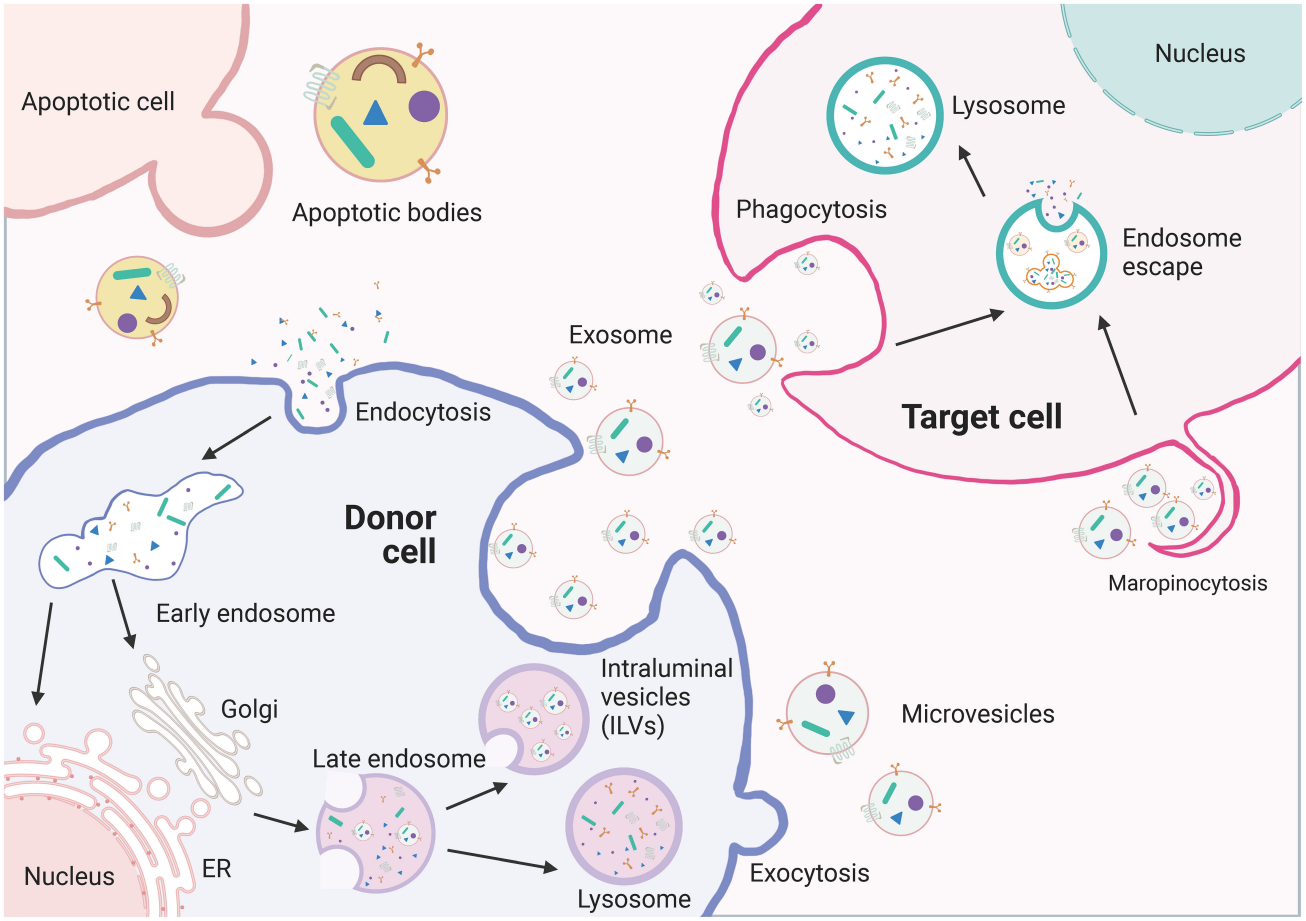
The biogenesis of extracellullar vesicles (EVs). EVs including exosomes, microvesicles, and apoptotic vesicles, are lipid bilayer particles that are naturally released from cells. The formation of EVs involves double invagination of the plasma membrane and intracellularization of intraluminal vesicles (ILVs). The formation of EVs involves the double invagination of the plasma membrane and the intracellular formation of intraluminal vesicles (ILVs), which are released into the extracellular space when MVBs fuse with the plasma membrane. However, microvesicles are produced by direct outward budding of the plasma membrane. The cargo contents of EVs are selectively packaged and depend on the maternal cell type and functional status. EVs can transfer bioactive RNAs, proteins, lipids, and metabolites from the donor cell to the recipient cell and influence the biological properties of the latter.

EVs are particles made up of lipid bilayer membranes that encapsulate the cytoplasmic sol compartment. They can be formed by outward sprouting of the plasma membrane or through the intracellular endocytic transport pathway, where multiple vesicles fuse with the plasma membrane in late-stage endocytic compartments (**Figure 4**). EVs can be categorized into exosomes, microvesicles, and apoptotic bodies based on their biochemical characteristics. Exosomes are generated through endosomal sorting complexes required for transport (ESCRT)-dependent or non-ESCRT-dependent pathways, which sort specific contents into exosomes (91, 92). Exosomes can fuse with the target cell membrane and deliver their cargo to the recipient cell through receptor-mediated endocytosis, megalocytosis, or phagocytosis (93, 94). Exosomes can also directly interact with target cells through receptor-ligand interactions for intercellular communication (95). Furthermore, the composition and biogenesis of exosomes can affect the condition of secretory cells and regulate the intra- and extracellular environment (96, 97).

### 3.2 The biological function of exosomes

Exosomes carry out diverse biological functions that depend on their cellular origin. The most crucial function of exosomes is signal transduction, as they transport molecules that regulate various physiological and pathological processes (98). As carriers of cellular communication, exosomes transport their substance to neighboring or distant cells, thereby regulating multifarious physiological and pathological procedure. Exosomes can also regulate the inflammatory vesicle activation. Stem cell-derived exosomes inhibit inflammatory vesicle activation, yet immune cell-derived exosomes trigger inflammatory vesicle activation, advising that exosomes maybe act as a therapeutic tool for diseases associated with inflammatory responses (99). Immune cell-derived exosomes also play a role in modulating immune responses, depending on the cellular environment in which they are produced (100, 101). Besides, exosomes have been implicated in neurovascular regeneration by promoting nerve regeneration and functional recovery through regulation of axon growth (102). They also serve as important biomarkers for disease diagnosis, particularly in the early detection of cancer (103). With the increasing research on exosomes, the role of them in tumor pathology has been continuously revealed. Tumor cell-secreted exosomes can contribute to the formation of a TME that make tumor cells evade immune surveillance and facilitate tumor growth (104). In addition, exosomes secreted by tumor cells can induce neoangiogenesis, which ensures access to nutrients and contributes to the continued proliferation of tumor cells (105).

### 3.3 The detection, quantification and identification methods of exosomes

Exosomes are dispersed in complex human body fluid specimens in a non-uniform membranous vesicle structure. Therefore, the separation and enrichment of exosomes is a key step in their detection technology. The purity and activity of the obtained exosomes can directly affect subsequent identification and functional analysis. At present, commonly used exosomes separation and enrichment techniques are mainly based on the physical and immunological characteristics of exosomes (106, 107). The centrifugation method separates exosomes by gradually increasing centrifugation force or time based on the differences in sedimentation coefficients of vesicles, cells, cell fragments, and protein molecules in solution (108). It mainly includes differential centrifugation and density gradient centrifugation. The precipitation method separates and purifies exosomes by co precipitation or reverse screening based on the physicochemical properties of compounds and exosomes (109). It mainly includes polymer precipitation method and organic solvent precipitation method. The particle size separation method separates exosomes based on the difference in particle size between them and other biomolecules, mainly including ultrafiltration and size exclusion chromatography (SEC) (110). Immunoaffinity methods use antigen-antibody interactions to specifically bind antibodies to exosomes (111). In addition, researchers have attempted to combine traditional techniques with microfluidic techniques based on the physicochemical characteristics of exosomes and developed new methods for exosomes separation, including capture microfluidic technology, filtration microfluidic technology, magnetic separation microfluidic technology, acoustic separation microfluidic technology, and dielectric electrophoresis microfluidic technology (112). Furthermore, the magnetic separation method based on aptamers involves modifying the surface of magnetic beads with aptamers, using them as recognition capture agents to specifically bind with exosomes surface proteins for exosomes capture (113). At present, there are more and more nucleic acid aptamer sensors for isolating exosomes, which have been successfully applied in the extraction of exosomes from samples such as urine, serum, and plasma.

The identification of exosomes involves analyzing their morphological characteristics, concentration, particle size, protein molecules, and cumulative components (114). Techniques like scanning electron microscopy, transmission electron microscopy, atomic force microscopy, and random optical reconstruction microscopy can be used to study the morphological features of exosomes (115, 116). Nanoparticle tracking analysis (NTA), dynamic light scattering (DLS), and tunable resistance pulse sensing technology provide real-time concentration and size analysis of exosomes (117). Flow cytometry is also applicable for particle number analysis of exosomes (118). Protein molecule characterization and concentration can be obtained through Western blot, ELISA, and flow cytometry (119). Topological identification focuses on exosomal membrane proteins and vesicular proteins and involves treatments, separations, proteomic analysis, verification, and analysis using techniques like protease treatment, biotinylation, liquid chromatography tandem mass spectrometry, mass spectrometry, flow cytometry, fluorescence microscopy, and bioinformatics.

## 4 Crosstalk between tumor cells and CAFs in GI tumors

Crosstalk between tumor cells and CAFs is crucial in the progression of GI tumors. Exosomes are believed to be involved in the transformation of GI tumors and act as signaling molecules between cells and can regulate tumor progression and suppression. They can target signaling pathways involved in tumor progression. CAFs can contribute to the resistance of GI tumors to therapy through various mechanisms. They can secrete exosomes that regulate signaling pathways and modulate the biological processes of GI tumor cells. Exosomes secreted by CAFs can restrain the biological processes of GI tumor cells, while GI tumor cells can promote the transformation of normal fibroblasts into CAFs through exosome-mediated interactions, thus creating a microenvironment favorable for tumor growth.

### 4.1 Crosstalk between cholangiocarcinoma and CAFs

CCA is a lethal tumor originating from epithelial cells in the bile ducts (120). CCA often presents at advanced stages, limiting surgical options (121, 122). Traditional radiotherapy is not very effective, and alternative treatments like molecular targeting and immunization require further exploration (123, 124). Studies have shown that CCA cell-derived exosomes can accelerate the malignant development of CCA by constraining the conversion of NFs to CAFs and thereby promoting the lethal progression of CCA. Qin et al (125), demonstrated that miR-34c expression was reduced in CCA cell exosomes, and exosomal miR-34c could be transferred to fibroblasts and mediate their activation. Mechanistic experiments indicate that miR-34c inhibits CCA progression by targeting WNT1, a gene involved in tumor growth. Similarly, decreased levels of miR-206 in intrahepatic CCA (iCCA) contribute to cell proliferation, migration, and invasion. Co-culturing CCA cells with NFs reduces miR-206 expression in NFs and promotes their transformation into CAFs. This interaction leads to increased aggression and resistance to gemcitabine in CCA cells. Overexpressing miR-206 either in CAFs or CCA cells suppresses this reciprocal promotion and inhibits tumor progression (126). However, further research is needed to fully understand the interaction between extracellular vesicles and CAFs in CCA, as well as explore potential therapeutic strategies targeting this interaction.

### 4.2 Crosstalk between HCC and CAFs

Surgical resection, liver transplantation, ablation, hepatic artery embolization and systemic chemotherapy, targeted drugs and immunotherapy are only effective for long-term survival in a limited number of patients (127, 128). Researches have shown that CAFs could restrain the biological processes of HCC cells through the secretion of exosomes (**Table 1**). Zhang et al (129), discovered that the expression level of miR-320a in CAFs-derived exosomes was obviously decreased in HCC and could be transported to HCC cells. Functional experiments revealed that exosomal miR-320a repressed the proliferation, migration and metastasis of HCC cells. Mechanistic experiments revealed that miR-320a could inhibit the activation of the MAPK pathway by targeting PBX3, and further inhibited the process of epithelial-mesenchymal transition (EMT), also the expression of cell-cycle protein-dependent kinase 2 (CDK2) and MMP2, thereby inhibiting cell proliferation and metastasis. Ultimately, this inhibits cell proliferation and metastasis. Therefore, CAFs delivering miR-320a to HCC cells could potentially be a therapeutic target for HCC. Yugawa et al (130), discovered that co-culturing HCC cells with conditioned medium from safranin-treated CAFs inhibits cell viability and invasiveness, while promoting apoptosis. Besides, the expression level of miR-150-3p was closely associated with the clinical characteristics of HCC patients. Lv et al (131), demonstrated that Safranin treatment also blocks the secretion of a circular RNA called circCCT3 in CAFs-derived exosomes. Besides, the expression of circCCT3 was significantly elevated in HCC, and treatment with safranin blocked the secretion of circCCT3 in the exosomes of CAFs. It is found that circCCT3 regulates glucose metabolism in HCC by affecting the expression of HK2. Liu et al (132), demonstrated the mechanism behind this involves the direct inhibition of DNA methyltransferase 3b (DNMT3b) expression by miR-29b, along with the up-regulation of metastasis suppressor 1 (MTSS1) expression, ultimately inhibiting the progression of HCC.

**Table 1:** Biological roles of exosomes between cancer cells and CAFs in HCC.

Donor cell	Recipient cell	Molecular	Expression	Biological function	Targets	Reference
CAFs	HCC	miR-320a	Down	Inhibit cells proliferation, migration, and metastasis	miR-320a/PBX3/MAPK/CDK/MMP2	(129)
		miR-150-3p	Down	Inhibit cells migration and invasion	/	(130)
		circCCT3	Up	Promote glucose metabolism	circCCT3/HK2	(131)
		miR-29b	Down	Inhibit cells growth and invasion	miR-29b/DNMT3b/MTSS1	(132)
		TUG1	Up	Promote cell migration, invasion, and glycolysis	TUG1/miR-524-5p/SIX1	(133)
		Gremlin-1	Up	Promote EMT and reduce sensitivity of sorafenib	Gremlin-1/Wnt/β-catenin/BMP	(134)
		miR-20a-5p	Up	Promote HCC malignant progression	miR-20a-5p/LIMA1	(135)
		circZFR	Up	Promote tumor growth and DDP resistance	circZFR/STAT3/NF-κB	(136)
HCC	CAFs	miR-1247-3p	Up	Promote the activation of CAFs	miR-1247-3p/B4GALT3	(137)

Researchers have found that exosomes derived from CAFs played a role in promoting aggressive behaviors in HCC cells (**Table 1**). These exosomes contain specific molecules that can accelerate cell migration, invasion, and metabolic reprogramming. For example, exosomal TUG1 identified by Lu et al., enhanced HCC progression by binding to miR-524-5p and upregulating SIX1 expression (133). Another study by Qin et al,. showed that exosomal Gremlin-1 from CAFs reduced the sensitivity of HCC cells to sorafenib and promotes epithelial-mesenchymal transition (EMT) by inhibiting the Wnt/β-catenin and BMP signaling pathways (134). Furthermore, Gremlin-1 was enriched in plasma exosomes of HCC patients and could predict the resistance to sorafenib in HCC patients. CAFs derived exosomes could also dramatically elevate the rates of intrahepatic and pulmonary metastasis of HCC. Jin et al (138), demonstrated that exosomes separated from CAFs could be absorbed by HCC cells and accelerated their invasive phenotype compared with para-cancerous fibroblasts (PAFs), and mechanistic experiments revealed that CAFs could offer multiple miRNAs (miR-329-3p, miR-380-3p, miR-410-5p, miR-431-5p) were delivered to HCC cells and increased their expression levels. Additionally, downregulation of LIMA1 expression was associated with poor OS and recurrence-free survival (RFS) in HCC patients. LIMA1 acted as a suppressor of HCC progression by inhibiting the Wnt/β-catenin signaling pathway. Treatment with miR-20a-5p-overexpressing CAFs exosomes inhibited LIMA1 expression and promoted aggressive tumor behavior in HCC (135). In addition, treatment of HCC cells with miR-20a-5p overexpressing CAFs exosomes inhibited LIMA1 expression and promoted lethal tumor progression *in vitro* and *in vivo*. Zhou et al (136), demonstrated that circZFR manifestation was boosted in cisplatin (DDP)-resistant HCC cells as well as in CAFs and CAFs-derived exosomes. Functional experiments revealed that circZFR could be obtained from CAFs cells to HCC cells via exosomes and accelerate tumor development and DDP resistance. Mechanistic experiments showed that circZFR could constrain the STAT3/NF-κB pathway and thus accelerate the progress of HCC and chemoresistance.

HCC cell-derived exosomes could also translocate into fibroblasts to promote a pro-tumorigenic microenvironment (**Table 1**). Fang et al (137), revealed that the exosome miR-1247-3p secreted by high metastatic HCC cells directly targetd B4GALT3, thereby activating β1-integrin-NF-κB signaling in fibroblasts. Activated CAFs further accelerated cancer progression by secreting pro-inflammatory cytokines, including IL-6 and IL-8. Clinical data showed that high levels of the serum exosome miR-1247-3p were associated with lung metastasis in HCC patients. Zhou et al (139), demonstrated that HCC cells had a strong ability to deliver common hematopoietic stem cells into CAFs. Similarly, exosomal miRNA-21 from HCC cells could be delivered to hematopoietic stem cells, which then transform into CAFs through the activation of the PDK1/AKT signaling pathway. These CAFs contributed to cancer progression through the secretion of angiogenic cytokines.

In terms of therapeutic applications, exosomes can be utilized as vehicles to deliver anticancer drugs for targeted treatment of HCC. However, more research is needed to fully understand the molecular mechanisms and targets involved in exosome-mediated drug delivery. Overall, further investigation is warranted to advance the translation of exosome-based technologies into clinical applications for HCC.

### 4.3 Crosstalk between CRC cells and CAFs

CRC is a deadly tumor that affects the GI tract and is a leading cause of cancer-related deaths worldwide. Currently, the primary examination modality for the primary diagnosis, screening and detection of CRC is colonoscopy (140). The main treatment modality for primary CRC is laparoscopic surgical treatment, and the main treatment modality for metastatic CRC is surgical resection combined with radiotherapy treatment (141). Despite advancements in screening and treatment, metastasis occurs in about 20% of patients with CRC and recurrence happens in about 40% of patients even with systemic treatment (142). Consequently, there has been growing interest in studying the interaction between CRC cells and other stromal cells in the TME.

#### 4.3.1 The effect of CRC cell-derived exosomes on CAFs

One aspect of this interaction is the impact of CRC cell-derived exosomes on CAFs. Exosomes derived from CRC cells have been shown to promote tumorigenesis and progression by influencing the polarization of fibroblasts, leading to the formation of CAFs, or by directly regulating CAFs (**Table 2**). Dai et al (143), discovered that miR-10b was enriched in exosomes of CRC cells and could be moved to fibroblasts compared with normal colorectal epithelial cells. Mechanistic experiments showed that exosomal miR-10b reduced fibroblast proliferation and accelerated the manifestation of TGF-b and SM a-actin by targeting and inhibiting the manifestation of PIK3CA and reducing the activity of the PI3K/Akt/mTOR pathway. This suggested that CRC cells could activate fibroblasts to become CAFs expressing myofibroblast markers by delivering the exosome miR-10b, which in turn promoted CRC growth. Yoshii et al (168), found that co-culture of fibroblasts *with TP53-deficient* HCT116 cells promoted their proliferative capacity compared with *in vitro* cultured *TP53-WT* HCT116 cells, and exosomes extracted from HCT116 cells after inhibiting the expression of TP53 in them inhibited TP53 manifestation in fibroblasts and facilitate their proliferation. The results of mechanistic experiments showed that the expression of miR-1249-5p, miR-6737-5p, and miR-6819-5p were significantly upregulated in TP53-deficient HCT116 cells, and they might be a novel therapeutic target for colon cancer. Noh et al (144), demonstrated that CRC cells could secrete exosomes into CAFs and accelerate the manifestation skill of let-7d in the cells, while let-7d could target and restrain the expression of CCL7 and thus inhibit the migration of THP-1 cells. Zhang et al (145), confirmed that exosomes from BAP31-regulated CRC affected the transport of common fibroblasts to pro-angiogenic CAFs. They found that BAP31 increased miR-181a-5p expression in CRC, and exosomal miR-181a-5p was also delivered to fibroblasts and significantly promoted angiogenesis in endothelial cells. Mechanistically, miR-181a-5p could directly target RECK and regulate the phosphorylation of MMP-9 and Smad2/3 to accelerate the transformation of fibroblasts into pro-angiogenic CAFs. Zhang et al (146), discovered higher grades of HSPC111 in serum exosomes, CAFs from main tumors and liver metastatic cancers patients in comparison with CRC patients without liver metastases. Besides, CRC cell-derived exosomal HSPC111 accelerated pre-metastatic niche formation and liver metastasis CRC (CRLM). Mechanistically, HSPC111 could alter lipid metabolism in CAFs by phosphorylating ACLY thereby upregulating acetyl-CoA levels, and acetyl-CoA accumulation further promoted CXCL5 expression and secretion by boosting H3K27 acetylation in CAFs. Furthermore, the CXCL5-CXCR2 axis could enhance CRC cell exocytosis of the exosome HSPC111, which in turn facilitated liver metastasis of CRC. Wang et al (147), discovered that miR-146a-5p and miR-155-5p expression was importantly increased in CRC cells with high CXCR7 expression and their exosomes. miR-146a-5p and miR-155-5p could be shifted from CRC cells to CAFs via exosomes and expressed in CAFs by regulating JAK2-STAT3/NF-κB signaling and targeting SOCS1 and ZBTB2 to accelerate the activation of CAFs, and the activated CAFs could enhance the intrusion capability of CRC cells. Mechanistic experiments showed that miR-146a-5p and miR-155-5p meaningfully increased the grades of inflammatory cytokines interleukin-6, tumor necrosis factor-α, transforming growth factor-β, and CXCL12 in CAFs, thereby triggering EMT and metastasis-promoting transformation of CRC cells. Besides, serum exosomal miR-146a-5p and miR-155-5p had the potential to be potential biomarkers for CRC.

**Table 2:** Biological roles of exosomes between cancer cells and CAFs in CRC.

Donor cell	Recipient cell	Molecular	Expression	Biological function	Targets	Reference
CRC	CAFs	miR-10b	Up	Inhibit the proliferation of fibroblasts and promote	miR-10b/PIK3CA/PI3K/Akt/mTOR	(143)
				the activation of CAFs		
		let-7d	Up	Inhibit THP-1 cell migration	let-7d/CCL7	(144)
		miR-181a-5p	Up	Promote the activation of CAFs	miR-181a-5p/RECK/MMP-9/Smad2/3	(145)
		HSPC111	Up	Modulate the lipid metabolism of CAFs	HSPC111/ACLY/acetyl CoA	(146)
		miR-146a-5p	Up	Promote the activation of CAFs	JAK2-STAT3/NF-κB/SOCS1-ZBTB2	(147)
		miR-155-5p	Up			
CAFs	CRC	circSLC7A6	Up	Promote cell proliferation and invasion and	circSLC7A6/CXCR5	(148)
				inhibit cell apoptosis		
		miR-17-5p	Up	Promote CRC metastasis	miR-17-5p/RUNX3RUNX3/MYC/TGF-β1	(149)
		LINC00659	Up	Promote cell proliferation, invasion and migration	LINC00659/miR-342-3p/ANXA2	(150)
		miR-135b-5p	Up	Promote tumor growth, cell proliferation, migration, and invasion,	miR-135b-5p/TXNIP	(151)
				inhibit cell apoptosis and promote HUVEC angiogenesis		
		circEIF3K	Up	Promote cell proliferation, invasion, and tube formation	circEIF3K/miR-214/PD-L1	(152)
		WEE2-AS1	Up	Promote cell proliferation and tumor formation	WEE2-AS1/MOB1A/Hippo	(153)
		miR-181b-3p	Up	Enhance cell proliferation and migration and reduce cell apoptosis	miR-181b-3p/SNX2	(154)
		miR-345-5p	Up	Promote CRC malignant progression	miR-345-5p/CDKN1A	(155)
		H19	Up	Promote the stemness and chemotherapy tolerance	H19/miR-141/β-catenin	(156)
		miR-92a-3p	Up	Promote cell stemness, EMT, metastasis,	miR-92a-3p/Wnt/-catenin/FBXW7/MOAP1	(157)
				and chemotherapy resistance		
		CCAL	Up	Inhibit cell apoptosis and promote OXA resistance	CCAL/HuR/β-catenin	(158)
		miR-93-5p	Up	Enhance cell proliferation and prevent	miR-93-5p/FOXA1/TGFB3	(159)
				radiation induced cell apoptosis		
		miR-24-3p	Up	Promote cell viability and colony formation,	miR-24-3p/CDX2/HEPH	(160)
				increase cell apoptosis, and promote MTX resistance		
		miR-590-3p	Up	Promote cell survival and tumor growth,	miR-590-3p/CLCA4/PI3K/Akt	(161)
				and enhance radiation resistance		
		METTL3	Up	Inhibit the sensitivity of 5-FU in CRC	METTL3/miR-181d-5p/NCALD	(162)
		cricN4BP2L2	Up	Promote the stemness and oxaliplatin resistance	cricN4BP2L2/EIF4A3/PI3K/AKT/mTOR	(163)
		miR-200b-3p	Down	Promote the sensitivity of 5-FU in CRC	miR-200b-3p/β-catenin/c-Myc	(164)
		miR-625-3p	Up	Promote cell migration, invasion, EMT,	miR-625-3/CELF2/WWOX	(165)
				and chemotherapy resistance		
		FOSL1	Up	Promote cell proliferation, stemness, and oxaliplatin resistance	FOSL1/ITGB4	(166)
		lnc-FAL1	Up	Inhibition of oxaliplatin induced cell autophagy	lnc-FAL1/TRIM3/Beclin1	(167)

Overall, understanding the crosstalk between CRC cells and CAFs can provide valuable insights into the mechanisms of CRC progression and potentially identify new therapeutic targets or biomarkers for the disease

#### 4.3.2 The effect of CAFs-derived exosomes on the growth and metastasis of CRC

The effect of CAFs-derived exosomes on the growth and metastasis of CRC was investigated in several studies (**Table 2**). Liu et al. (169) showed that CAFs-derived exosomes enhanced the clonogenicity and radio-resistance of CRC cells by activating the TGF-β signaling pathway. Gu et al. (148) found that circSLC7A6, highly expressed in CRC tissues, promoted cell proliferation and invasion, but its effects were inhibited by the drug matrine. Zhang et al (149), discovered that miR-17-5p expression in CAFs-derived exosomes was higher in CRC than in normal fibroblasts, and the transfer of miR-17-5p via exosomes promoted CRC metastasis. Zhou et al (150), observed that CAFs-derived exosomes accelerated proliferation, migration, invasion, and EMT progression of CRC cells by delivering LINC00659 to CRC cells. Yin et al (151), revealed that miR-135b-5p, enriched in CAFs exosomes, promoted CRC cell growth, migration, invasion, and angiogenesis by targeting TXNIP. Yang et al (152), found that hypoxia induced the secretion of circEIF3K from CAFs exosomes, which enhanced CRC cell proliferation, invasion, tube formation, and tumor growth. Yang et al (153), demonstrated that WEE2-AS1 was significantly higher in plasma EVs of CRC patients than in healthy subjects, and its high grade predicted progressive pathological stage and low survival rate. In addition, WEE2-AS1 expression was significantly elevated in CAFs-derived sEVs and could be delivered to CRC cells to accelerate cell proliferation as well as tumor formation and progression *in vivo*. Mechanistically, WEE2-AS1 interflowed with MOB1A and accelerated MOB1A degradation, which in proper order promoted CRC cell growth by inhibiting the Hippo pathway. Jiang et al (154), revealed that miR-181b-3p was abundant in exosomes of CAFs and could be transferred into CRC cells and increased the expression of miR-181b-3p. Functional researches revealed that exosomal miR-181b-3p significantly contributed to the proliferation and migration of CRC cells and significantly diminished apoptosis. The consequence of mechanistic experiments revealed that exosomal miR-181b-3p could regulate the expression of SNX2. Shi et al (155), identified that miR-345-5p expression was significantly elevated in CAFs-exo compared with NFs-exo, and CAFs-exo could reconcile the move of miR-345-5p into CRC cells to redound lethal tumor progression. Mechanistically, miR-345-5p could forthrightly target CDKN1A to promote CRC progression and metastasis.

#### 4.3.3 The effect of CAFs-derived exosomes on the therapy of CRC

CAFs have been found to influence the therapeutic outcomes of CRC through the release of exosomes (**Table 2**). Ren et al (156), discovered that the expression of H19, a long non-coding RNA, was significantly higher in tumor tissues of colitis-associated carcinoma (CAC) mice and CRC patients. They found that H19 was enriched in CAF-derived exosomes and could be transmitted to CRC cells, where it regulated the β-catenin pathway by binding to miR-141. This resulted in the promotion of cancer stem cell properties and chemotherapy tolerance in CRC cells. Furthermore, CAF-derived exosomes were found to enhance tumor growth and reverse the antitumor effects of the chemotherapy drug oxaliplatin (OXA). Hu et al (157), demonstrated that miR-92a-3p, enriched in CAF-derived exosomes, could be delivered to CRC cells where it accelerated cancer stemness, EMT, metastasis, and chemoresistance. This was mediated through the activation of the Wnt/β-catenin pathway and inhibition of mitochondrial apoptosis by directly suppressing FBXW7 and MOAP1. Exosomal miR-92a-3p was also found to be abundant in the serum of CRC patients and associated with metastasis and chemoresistance. Additionally, CAF-derived exosomes promoted the formation of lung metastasis nodules. Deng et al (158), found that CCAL expression was higher in the tumor stroma compared to the cancer nests of CRC tissues. CCAL, transported from CAFs to CRC cells via exosomes, promoted OXA resistance, inhibited apoptosis, induced chemoresistance, and activated the β-catenin pathway. This was achieved through the direct interaction of CCAL with mRNA, leading to the moderation of protein HuR and increased β-catenin expression. Chen et al (159), demonstrated that miR-93-5p expression in CAF-derived exosomes was significantly higher compared to that of NFs. Exosomal miR-93-5p enhanced CRC cell proliferation, protected cells from radiation-induced apoptosis, and promoted tumor growth. Mechanistically, miR-93-5p targeted FOXA1, which was connected to the promoter of TGFB3 and inhibited the nuclear accumulation of TGFB3. This suggested that miR-93-5p could serve as a potential therapeutic target for preventing CRC cells from resisting radiation-induced apoptosis. Zhang et al (160), found that miR-24-3p was highly expressed in both colon cancer (CC) tissues and cells. CAF-derived exosomal miR-24-3p accelerated cell viability, colony formation, and apoptosis through down-regulation of the CDX2/HEPH axis. It also promoted methotrexate (MTX) resistance in CC cells and enhanced CC resistance to MTX *in vivo*. Chen et al (161), demonstrated that miR-590-3p expression was significantly increased in CRC tissues and cell lines. CAF-derived exosomal miR-590-3p was delivered to CRC cells, promoting cell survival and tumor growth. Mechanistically, miR-590-3p enhanced radio-resistance in CRC by regulating the CLCA4/PI3K/Akt signaling pathway. Pan et al (162), found that m6A modification and METTL3 expression were upregulated in CRC patients. METTL3 facilitated m6A methylation modification of miR-181b-5p, derived from DGCR8 in CAFs. CAF-derived exosomes inhibited the sensitivity of CRC cells to 5-FU through the METTL3/miR-181d-5p axis, where miR-181d-5p directly targeted NCALD. Qu et al (163), discovered that CAF-derived exosomal circular RNA cricN4BP2L2 could be transferred to CRC cells and promote OXA resistance and stemness while inhibit apoptosis. CricN4BP2L2 could inhibit the PI3K/AKT/mTOR signaling axis through the interaction with EIF4A3. Yuan et al (164), found that the expression of miR-200b-3p was significantly decreased in CRC tissues compared to normal neighboring tissues. Additionally, the expression of miR-200b-3p in exosomes derived from hypoxic CAFs was lower compared to normoxic conditions. Functional experiments showed that exosomes from hypoxic CAFs with up-regulated miR-200b-3p increased CRC cell sensitivity to 5-fluorouracil (5-FU) both *in vitro* and *in vivo*. The mechanism involved the regulation of the β-catenin/c-Myc axis, thereby promoting the progression of CRC by targeting HMBG3. Zhang et al (165), demonstrated that exosomes released by CAFs could be taken up by CRC cells. Exosomal miR-625-3p derived from CAFs enhanced the invasion, migration, EMT and chemoresistance of CRC cells by regulating the CELF2/WWOX pathway. Moreover, exosomal miR-625-3p could promote tumor growth. Shi et al (170), showed that exosomes derived from chemo-resistant CAFs (R-CAFs) promoted CRC cell viability, inhibited apoptosis, and induced angiogenesis. These exosomes transferred to HT29 cells accelerated CRC development and resistance to the chemotherapy drug DDP by upregulating VEGFA expression. Lin et al (166), demonstrated that the expression of FOSL1 and ITGB4 was crucial in CRC progression and predicted poor prognosis. However, only FOSL1 was found to be enriched in CAFs. Exosomes derived from CAFs transported FOSL1 to CRC cells, which then transcriptionally activated ITGB4 to accelerate CRC cell proliferation, stemness, and resistance to the chemotherapy drug oxaliplatin. Zhu et al (167), discovered that the lnc-FAL1 was significantly overexpressed in CRC samples, indicating a poor prognosis. Additionally, lnc-FAL1 was highly enriched in CAF-derived exosomes and could inhibit OXA-induced autophagy in CRC cells. The mechanism involved lnc-FAL1 acting as a scaffold to promote TRIM3-dependent Beclin1 polyubiquitination and degradation, thereby inhibiting autophagic cell death induced by OXA.

Currently, research on the relationship between EVs, CAFs, and CRC cells is still limited to basic experimental studies, with few clinical cases reported. Translating the results obtained from cellular and animal models into clinical trials remains a significant challenge.

### 4.4 Crosstalk between ESCC cells and CAFs

Crosstalk between ESCC cells and CAFs has been observed to play a significant role in the development and progression of ESCC (171). Even after undergoing extended radical resection, the 5-year OS rate of patients with clinically high-level ESCC remains poor (6). Studies have revealed that there are also mutual efficacy between ESCC cells and CAFs. Zhao et al (172), confirmed that the manifestation levels of SHH were importantly elevated in CAFs lysate, conditioned medium for culturing CAFs (CAF-CM) and CAFs-derived exosomes. Hedgehog signaling pathway has been found to be promoted in ESCC cells when treated with CAF-derived factors such as conditioned medium and exosomes. This stimulation of the Hedgehog pathway leads to increased cell growth and migration. Jin et al (173), discovered that the expression level of hsa-miR-3656 has been found to be higher in CAFs compared to NFs. Overexpression of miR-3656 in CRC cells also enhances cell proliferation, migration, and invasion. Exosomal miR-3656 can be transferred from CAFs to ESCC cells, where it activates the PI3K/AKT and Wnt/β-catenin signaling pathways by down-regulating ACAP2. This activation ultimately promotes ESCC growth and metastasis. Shi et al (174), confirmed that LINC01410 was enriched in CAFs and could be transferred to ESCC cells and increase their LINC01410 levels. This transfer of LINC01410 promoted ESCC metastasis and EMT by binding to miR-122-5p, resulting in increased PKM2 levels. Cui et al (175),. demonstrated that CAFs-derived exosomes might accelerate cell proliferation and regulate apoptosis by RIG-I/IFN-β signaling and influence the chemosensitivity of ESCC to DDP, and that targeted inhibition of the RIG-I/IFN-β signaling axis in the exosomes of CAFs maybe an underlying therapeutic tactics for ESCC. Furthermore, the presence of CAFs and micro-lymphatic vessels in ESCC was associated with a poor prognosis (176). Exosomes derived from CAFs promote invasion, proliferation, migration, and tube formation of tumor-associated lymphatic endothelial cells (TLECs), while miR-100-5p exerts the opposite effect. Mechanistically, miR-100-5p inhibited ESCC lymphatic metastasis by targeting the IGF1R/PI3K/AKT regulatory axis and suppressing lymphangiogenesis.

In addition, ESCC cell-derived exosomes also regulated the conversion of NFs to CAFs. Interestingly, ESCC cell-derived exosomes have also been found to play a role in the conversion of NFs into CAFs (177). The transfer of lncRNA POU3F3 from ESCC cells to NFs leads to their transformation into CAFs. These activated fibroblasts further enhance ESCC cell proliferation and resistance to cisplatin by secreting interleukin 6 (IL-6). Moreover, high levels of plasma exosomal lncRNA POU3F3 are associated with poor treatment response and survival outcomes in ESCC patients. While it has been established that CAFs and ESCC cells communicate and regulate tumor development, progression, and drug resistance through exosomes, the specific mechanisms involved require further exploration and research due to limited current understanding.

### 4.5 Crosstalk between GC cells and CAFs

Crosstalk between GC cells and CAFs has been a focus of research due to the challenges in GC occurrence, progression, and treatment (178, 179). The median survival of patients with metastatic or unresectable GC is still less than two years (180). Finding the difficulties in GC occurrence and progression as well as clinical treatment has been the direction of research in the last few years. Studies have shown that CAFs could regulate GC progression through exosomes (**Table 3**). Miki et al (188), certified that exosomes carrying CD9 were taken up by GC cells, promoting their migration and invasion. Patients with CD9-positive GC were associated with worse clinical outcomes. *In vitro* cellular assays showed that exosomes from CAFs promoted the migration and invasion of OCUM-12 and NUGC-3 cells, which was regulated by anti-CD9 antibody or CD9-siRNA. In addition, CD9-positive GC patients were significantly associated with smooth muscle-type GC, lymph node metastasis and venous invasion, and had a meaningfully lower 5-year survival rate. Xu et al (181), confirmed that MMP11 was enriched in CAFs and could be separated to GC cells thereby promoting the facilitation of GC cell migration. Besides, MMP11 was significantly overexpression in exosomes refined from both plasma and tumor tissues of GC patients and was strongly relevant to poor prognosis of patients. The consequence of mechanistic experiments revealed that exosomal miR-139 could regulate GC growth and metastasis by decreasing the expression of MMP11. Zhang et al (182), demonstrated that exosomal miR-522 was mainly separated from CAFs of GCs, and USP7 could mediate the entry of miR-522 into exosomes by deubiquitinating and stabilizing hnRNPA1. In addition, they identified that DDP and paclitaxel could accelerate miR-522 secretion from CAFs by activating the USP7/hnRNPA1 axis and reduce the gross of lipid-ROS in GC cells by restraining ALOX15, which finally reduced chemosensitivity *in vitro* and *in vivo*. Overall, the exosomal miR-522 secreted by CAFs could inhibit iron apoptosis in GC cells by targeting ALOX15 and blocking lipid-ROS accumulation. Shi et al (183), discovered that CAFs could deliver functional circ_0088300 to GC cells via exosomes and promote cell migration, proliferation and invasive capacity. Mechanistically, KHDRBS3 could transport circ_0088300 packaging into exosomes, and circ_0088300 could act as a sponge to target miR-1305 and inhibit the JAK/STAT signaling pathway to accelerate the proliferation, migration and intrusion of GC cells. Wang et al (184), identified that miR-199a-5p could be transferred into GC cells via CAFs-derived EVs and promoted the malignant phenotypes and gastric tumorigenesis *in vitro* and *in vivo*. Mechanistically, miR-199a-5p could increase the phosphorylation level of AKT1 by down-regulating FKBP5 thereby promoting the lethal phenotype of AGS cells through mTORC1. Qu et al (185), demonstrated that DACT3-AS1 expression was reduced in GC and associated with low prognosis in GC patients, and the results of *in vitro* and *in vivo* experiments revealed that DACT3-AS1 inhibited cell proliferation, migration and invasion by targeting the miR-181a-5p/sirtuin 1 (SIRT1) axis. In addition, DACT3-AS1 was mainly delivered from CAFs to GC cells via exosomes and could attenuate the growth of xenograft tumors. Furthermore, DACT3-AS1 could sensitize cancer cells to oxaliplatin via SIRT1-mediated iron oxidation.

**Table 3:** Biological roles of exosomes between cancer cells and CAFs in GC.

Donor cell	Recipient cell	Molecular	Expression	Biological function	Targets	Reference
CAFs	GC	MMP11	Up	Promote cells proliferation and migration	miR-139/MMP11	(181)
		miR-522	Up	Reduce chemotherapy sensitivity	USP7/hnRNPA1/miR-522/ALOX15	(182)
		circ_0088300	Up	Promote cells proliferation, migration and invasion	KHDRBS3/circ_0088300/miR-1305/JAK/STAT	(183)
		miR-199a-5p	Up	Promote GC malignant progression	miR-199a-5p/FKBP5/AKT1/mTORC1	(184)
		DACT3-AS1	Down	Inhibit tumor growth and increased sensitivity of oxaliplatin	DACT3-AS1/SIRT1	(185)
GC	CAFs	miR-27a	Up	Promote cell proliferation and migration,	/	(186)
				and the activation of CAFs		
		LINC00691	Up	Promote the activation of CAFs	LINC00691/JAK2/STAT3	(187)

In addition, GC cells also function to regulate CAFs (**Table 3**). Wang et al (186), discovered that miR-27a enticed programming coding of fibroblasts into CAFs. Besides, they demonstrated that miR-27a was enriched in the exosomes of GC cells and could promote the proliferation and motility of GC *in vitro* and *in vivo* by targeting CSRP2. Xia et al (187), confirmed that the expression of LINC00691 in serum exosomes of GC patients was higher above the fine subjects and patients with benign gastric disease and correlated with the clinicopathology of GC patients. Treatment of NFs with GC exosomes increased their LINC00691 expression levels and enabled them to acquire the properties of CAFs. Mechanistic experiments date revealed that LINC00691 could inhibit the JAK2/STAT3 signaling pathway to convert NFs into CAFs. The value of exosomes in the clinical diagnosis, treatment, and prediction of recurrence and metastasis of GC needs further confirmation to truly translate into clinical practice.

### 4.6 Crosstalk between PC cells and CAFs

PC is a highly lethal GI tumor with an adding incidence, clinically characterized by difficulty in early diagnosis, poor rate of surgical resection, high rate of postoperative recurrence, and low prognosis, with the global five-year survival rate of patients being only about 10% (189, 190). Research finding have revealed that CAFs could be separated to PC cells via exosomes to inhibit cell pullulation and metastasis (**Table 4**). Zhao et al (196), demonstrated that miR-320a expression was significantly increased in exosomes derived from CAFs compared to those from NFs. These miR-320a-enriched exosomes could transfer miR-320a to macrophages and promote M2 polarization, which, in turn, accelerated PC cell proliferation and invasion. Mechanistic experiments revealed that miR-320a regulated the PTEN/PI3Kc signaling pathway to promote the lethal progression of PC. Zhou et al (191), demonstrated that treatment of PC cells with CAF exosomes increased the expression of intracellular miR-421, leading to enhanced cell migration, proliferation, and invasion. Mechanistic experiments revealed that miR-421 targeted SIRT3 and regulated H3K9Ac, resulting in increased expression of HIF-1α. These findings suggested that miR-421 could be transferred from CAFs to PC cells via exosomes, promoting the development of PC. Raghavan et al (197), confirmed that secreted EVs derived from a specific subtype of CAFs called NetG1^+^ CAFs were found to stimulate Akt-mediated survival in nutrient-poor PDAC cells, protecting them from apoptosis. The expression of NetG1 in CAFs was identified as necessary for the pro-survival properties of EVs. Guo et al (192), demonstrated that miR-125b-5p expression was significantly boosted in PC cell lines and tissues. Besides, miR-125b-5p was enriched in the exosomes of CAFs and could be separate to PDAC cells to accelerate PDAC growth, invasion and metastasis by targeting APCs.

**Table 4:** Biological roles of exosomes between cancer cells and CAFs in PC.

Donor cell	Recipient cell	Molecular	Expression	Biological function	Targets	Reference
CAFs	PC	miR-421	Up	Promote cell proliferation, migration and invasion	miR-421/SIRT3/H3K9Ac/HIF-1α	(191)
		miR-125b-5p	Up	Promote tumor growth, invasion and metastasis	miR-125b-5p/APC	(192)
		miR-106b	Up	Promote the resistance of GEM	miR-106b/TP53INP1	(193)
		miR-21	Up	Promote cell proliferation and resistance of chemotherapy	miRNAs-PTEN	(194)
		miR-181a				
		miR-221				
		miR-222				
		miR-92a				
		miR-3173-5p	Up	Promote the resistance of GEM	miR-3173-5p/ACSL4	(195)

In addition, CAFs-derived exosomes have a vital regulatory function in the clinical management of PC (**Table 4**). Richards et al (198), identified CAFs exposed to chemotherapy could promote PC cell survival and amplification. Besides, CAFs exposed to GEM had increased exosomal release and accelerated cell amplification and drug resistance through delivery to passaged snail in receptor epithelial cells. Kong et al (193), isolated CAFs from primary fibroblasts of PC patients and discovered that CAFs were congenitally oppose to GEM by assay. Treatment of CAFs or CAFs-exosomes using GEM could promote the effect of PC cell proliferation. The results of mechanistic experiments discovered that miR-106b could be directly moved from CAFs to PC cells via exosomes and promoted the opposition of PC cells to GEM by directly targeting TP53INP1. Fang et al (194), found that miR-21, miR-221, miR-181a, miR-222, and miR-92a expression were elevated in CAF exosomes secreted during gemcitabine treatment and could promote cell amplification and chemoresistance by inhibiting PTEN. Treatment with the inhibitor GW4869 could block the inhibition of PTEN *in vivo* and thus improve the chemotherapeutic efficacy of PDAC. Qi et al (195), confirmed that CAFs would be separated to PDAC cells via secreted exosomes and accelerate chemoresistance after GEM treatment. Automatically, miR-3173-5p in CAF exosomes targets ACSL4 and suppresses iron mutations in PDAC. In conclusion, the exosomal miR-3173-5p/ACSL4 pathway might be a promising target for the treatment of GEM-resistant PC. Exosomes, as carriers and messengers of intercellular cross talk, play a regulatory role in the occurrence, development, drug resistance and tumor immunity of PC. Although the underlying mechanisms are not yet fully understood, the abundant biological information contained in exosomes provides opportunities for uncovering the pathogenesis of PC and developing targeted therapeutic interventions.

## 5 Discussion and prospects

GI tumors are a significant health threat with a high incidence rate. However, early clinical symptoms are often atypical and the diagnosis rate is low, resulting in most patients being diagnosed at a progressive or advanced stage. Even if surgery is performed at this stage, the outcomes and prognosis are often unsatisfactory. The treatment of early GI tumors is primarily based on radical surgery, while progressive tumors require a combination of radiotherapy and chemotherapy. However, chemotherapy resistance and intolerance to radiotherapy lead to poor prognosis in patients.

Extensive and multilevel interchange between tumor cells and mesenchymal cells offer the TME with conditions that sustain tumor survival, development, and metastasis (73, 199). CAFs are abundant and actively participate in tumor progression through interactions with immune cells and tumor cells. These interactions occur via various signaling pathways, such as autocrine and paracrine communication, forming a complex molecular network that influences the biological functions of the TME (74, 200), forming a complex molecular network and thus exerting its biological functions. Compared with other types of EVs, the structure of exosomes is more stable and the contents that can be encapsulated are more abundant, making them excellent drug delivery carriers, and the rich contents make them have diagnostic potential (201–203). Exosomes, a type of extracellular vesicle, have emerged as important mediators of intercellular communication within the TME. Exosomes can influence tumor angiogenesis, tumor aggressiveness and drug resist tumor metabolic reprogramming and immune escape through interactions with the tumor microenvironment (204, 205). Meanwhile, since exosomes are membranous structures, their details are difficultly divided rank by extracellular proteases and are highly stable in body fluids (205, 206). They exhibit greater stability and can encapsulate a wide range of contents, making them ideal carriers for drug delivery. Additionally, the rich contents of exosomes make them potential diagnostic markers for early cancer detection, prognosis prediction, and treatment response assessment (207, 208), and the restraining efficacy of exosomes on tumor growth and drug resistance can also be utilized to design targets for therapeutic purposes (209–211). In addition, exosomes have immune regulatory functions that can regulate the body’s immune response and provide new ideas for the treatment of immune related diseases (202). It can also promote tissue regeneration and repair, providing new therapeutic strategies for regenerative medicine and tissue engineering (212). Furthermore, exosomes can be utilized for personalized treatment. By tailoring drugs and treatment plans based on each patient’s specific condition, treatment effectiveness can be improved, leading to greater patient satisfaction.

Exosomes of GI tumor cell origin can deliver bioactive substances between cells and have a vital function in the incident and development of GI tumors. The stability brought by exosomes and the differences in expression profiles of macromolecular contents make them have good application prospects in inchoate diagnosis and prognosis judgment of GI tumors, and liquid biopsy based on them is promising to turn into an important approach of tumor diagnosis in the future. For example, circulating exosomal lncRNA-GC1 can be used as a standalone prognostic predictor of disease-free and overall survival in GC, with better predictive precision of disease-free and overall survival compared with the conventional AJCC staging system alone (C index: DFS 0.701 vs 0.614; OS 0.720 vs 0.611) (213). Five piRNAs in serum exosomes from HCC can forcefully confirm HCC patients from non-tumor donors based on regions under the Acceptor Operating Characteristics (AUROC) model, and can also be valuable for the diagnosis of HCC with poor tumor encumbrance (214). In addition, in GI tumor therapy, exosomes can be used as antitumor drug targeting carriers, tumor control targets, taking office of antitumor immunity, and creation of new therapeutic strategies for patients who are resistant to clinical chemotherapy and cannot tolerate chemotherapy (215, 216).

Recent research has shown that CAFs play a crucial role in the growth of gastrointestinal tumors. CAFs and tumor-derived exosomes have important biological functions in tumorigenesis and development, influencing each other’s functions. Additionally, the mechanisms of exosome formation and regulation between tumor cells and CAFs, the similarities and differences in their components, and cell-specific communication between exosomes in the microenvironment need further investigation. Current clinical treatments often involve inhibiting relevant genes or the packaging process of target exosomes, but these methods lack precision. Interfering directly with exosome release can have unintended consequences on the transport of other molecules. Exosomal release from CAFs can inhibit the development and metastasis of gastrointestinal tumors and reduce resistance to chemotherapy. High risk factors can induce unique molecular over-expression in exosomes of GI cancers. The cytotoxin associated gene A (CagA) of Hp can reduce the immunotherapeutic effect of Hp infection on GC by inhibiting the proliferation and anti-cancer effects of p53 and miR-34a, as well as CD8^+^T cells, and increasing the level of PD-L1 in exosomes derived from GC cells (217). In addition, Hp can induce upregulation of MET in GC cell exosomes and enhance the pro tumor effect of tumor associated macrophages (218). Hepatitis virus can promote the upregulation of various components in tumor extracellular vesicles, thereby promoting the occurrence and malignant progression of liver cancer (219–224). Interventions targeting these high-risk factors can achieve therapeutic effects by affecting the secretion profile of cancer cell exosomes. At the same time, CAFs are donors of target genes, and many exosomes secreted by GI tumor cells also require the participation of CAFs to exert their effects. Therefore, the identification of optimal targets in different kinds of tumors and their tumor-associated cells is of great significance and the biggest challenge for the application of exosomes in the clinic. GI-cancers have some unique characteristics compared to other types of cancers, PC and one subtype of GCa have plenty stromal cells but a few tumor cells (~10%), which presents the difficulty in drug delivery and chemoresistance. CAFs are the most important component of the stroma in digestive tract tumors, with significant heterogeneity in their sources and functions. GI tumor cells can recruit and activate CAFs through cross talk, jointly reshaping TME and ultimately affecting the malignant phenotype and chemotherapy resistance of tumor cells. Targeting CAF/exosome may provide a novel therapeutic approach to these cancers. Meanwhile, deep research of targets in CAFs is not only for designing therapeutic approaches against the targets, but also for further understanding the instinct and biological characteristics of CAFs and would help to explore other therapeutic approaches against CAFs.

Currently, there is limited clinical research on exosomes, mainly focusing on verifying their diagnostic effectiveness (225–227). While exosomes hold potential value in clinical diagnosis and treatment, they are still in the exploratory stage and require further research and validation. The lack of a standardized process for extracting and detecting extracellular vesicles leads to variations in methods used by different research teams, resulting in discrepancies in results and difficulties in making comparisons. This lack of standardization also hampers the application of extracellular vesicles as biomarkers in clinical diagnosis and treatment. Furthermore, exosomes display significant heterogeneity, varying in content, source, and function. This heterogeneity is influenced by factors like cell types, differentiation states, and stimulation conditions. Consequently, accurately identifying and isolating exosomes with specific functions remains challenging in clinical trials. Despite the promising outcomes observed in clinical trials, translating these findings into practical applications poses a challenge. This involves optimizing the preparation and purification methods of extracellular vesicles, establishing standards for them as biomarkers, and designing effective treatment plans. Moreover, there are several limitations in *in vivo* experiments involving exosomes. Animal models may differ significantly from human physiological and disease processes, making the results less applicable to humans. Animals may mount an immune response to exosomes, leading to deviations in experimental outcomes. The complex separation and purification process of exosomes in animal experiments makes it difficult to ensure their stability and activity. Multiple factors can influence the results observed in animal models, making the interpretation of experimental outcomes more complex. Additionally, ethical issues and societal controversies surround the use of animals in experiments, which also results in higher experimental costs in terms of human, material, and financial resources. Consequently, when exosomes with promising animal experimental results progress to the clinical trial stage, their efficacy and safety may not be guaranteed, increasing the risk and uncertainty in drug development. Nonetheless, as research continues, leveraging the advantages of exosomes as natural carriers holds potential, and significant progress is expected in exosome-based cancer treatment strategies.

## 6 Conclusion

In conclusion, the role of exosomes in CAFs and GI tumor cells is significant. CAF-derived exosomes contribute to the acceleration of GI tumor progression and drug resistance, while exosomes from GI tumor cells activate common fibroblasts to adopt CAF-like phenotypes. This process forms a network of knowledge exchange between CAFs and GI tumor cells, playing a meaningful role in GI tumor progression, metastasis, and drug resistance. This review has provided a summary of recent research progress on exosomes in CAFs and GI cancer cells, aiming to offer new insights for further investigation into the function of CAFs in GI cancers.

## Author contributions

LC: Writing – original draft, Writing – review & editing. HO: Writing – original draft, Writing – review & editing.
